# Early life impacts of maternal obesity: a window of opportunity to improve the health of two generations

**DOI:** 10.1098/rstb.2022.0222

**Published:** 2023-09-11

**Authors:** Laura Dearden, Susan E. Ozanne

**Affiliations:** University of Cambridge, Wellcome MRC Institute of Metabolic Science, University of Cambridge, Cambridge, CB2 0QQ, UK

**Keywords:** obesity, diabetes, pregnancy, developmental programming

## Abstract

The number of pregnancies complicated by obesity is increasing in line with the worldwide obesity crisis; recent estimates suggest that in developed countries more than 50% of pregnancies are in women who are overweight or have obesity. Maternal obesity is associated with an increased risk of many adverse outcomes for both the mother and baby during pregnancy and birth. In addition to these immediate outcomes, maternal obesity before and during pregnancy is associated with an increased risk of offspring cardio-metabolic disease later in life. Studies comparing siblings discordant for *in utero* exposure to maternal obesity suggest this is not simply due to transmission of ‘obesogenic genes’ between mother and child or current lifestyle factors, but reflects a direct impact of the obese intrauterine environment on fetal development. This review will describe the long-term consequences of exposure to maternal obesity during development for the cardio-metabolic health of the offspring. It will also discuss the potential molecular mechanisms that underlie the increased risk of metabolic disease in offspring of mothers with obesity, and explore interventions that may be implemented during pregnancy to limit the impact of obesity on offspring long-term health.

This article is part of a discussion meeting issue ‘Causes of obesity: theories, conjectures and evidence (Part I)’.

## 1. Introduction

In recent decades worldwide obesity levels have increased exponentially. Genetic variation is one important source of variation in body mass index (BMI); effect sizes range from genome-wide association studies for common genetic polymorphisms that explain around 10% of the heritability of the condition [1], to recent studies combining both common and rare genetic forms of genetic variation that explain 30% of BMI variation [2]. Twin and family studies estimate the total heritability of BMI to be between 40 and 75% [3,4]. However interpretation of such studies are complicated by the complexities of accounting for shared fetal environment—70% of monozygotic twins share a placenta—and current environment (and the interaction of both with genotype—see §2). The incomplete explanation of BMI by genetics shows that there is a strong impact of the environment on obesity susceptibility. The environment that prevails today—in which we live increasingly sedentary lifestyles and have easy access to high-calorie, ultra-processed foods—undoubtedly contributes to rising obesity rates. However, evidence suggests the environment experienced by an individual during their early life (from around conception to age two) also influences long-term obesity risk, a concept embodied within the Developmental Origins of Health and Disease (DOHaD). An association between the fetal environment and later incidence of metabolic disease was first reported by Hales and Barker, who proposed the ‘thrifty phenotype hypothesis' based on their observations of a link between reduced birth weight and increased cardio-metabolic disease in adulthood [5,6]. Further studies examining individuals who were *in utero* during the Dutch Hunger Winter (from 1944 to 1945) confirmed an association between *in utero* under-nutrition and the development of metabolic disease, and suggested it was a causative relationship [7].

**Figure 1.**
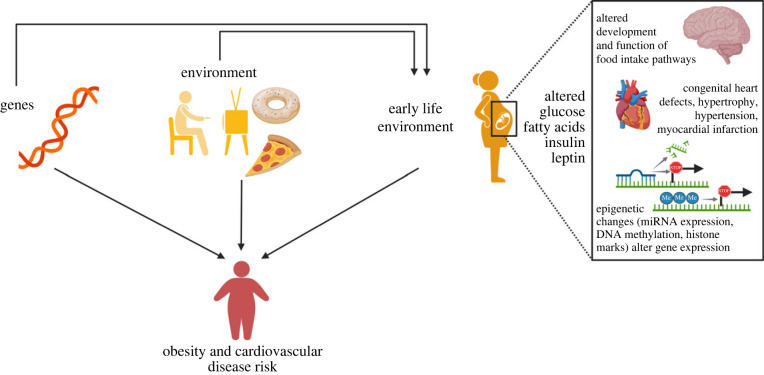
Genetic factors and the environment contribute to an individual's risk of developing obesity and cardiovascular disease. These factors can act directly or via maternal health in pregnancy, creating a sub-optimal *in utero* environment that increases susceptibility to develop cardio-metabolic disease. This increased disease risk is caused by changes in hormone or nutrient levels that directly affect the development and later function of organs such as the brain and heart, or via epigenetic mechanisms that result in permanent changes to gene expression.(Online version in colour.)

Although DOHaD was originated from observations of fetal growth restriction and undernutrition *in utero*, the more common situation nowadays is that of a mother being overweight or having obesity during pregnancy. The number of pregnancies complicated by obesity is increasing; recent figures show that in developed countries more than 50% of pregnancies are in women who are overweight or have obesity [8,9], and of these up to a third are also complicated by gestational diabetes mellitus (GDM) [10]. Furthermore, recent reports suggest that in the US, up to 40% of pregnant women exceed the Institute of Medicine guidelines for recommended gestational weight gain (GWG) during pregnancy [11]. An increasing amount of evidence shows that maternal obesity before and during pregnancy is associated with an increased risk of offspring cardio-metabolic disease. Studies comparing siblings discordant for *in utero* exposure to maternal obesity or GDM suggest this is not simply due to transmission of ‘obesogenic genes’ or shared lifestyle factors. Studies of siblings born before and after their mother underwent weight reducing gastric bypass surgery show children born from the pregnancy when the mother was lean have reduced adiposity and improvements in insulin sensitivity compared to their siblings who were born from a pregnancy when the mother had obesity [12]. Similarly, in siblings discordant for exposure to GDM, the sibling who was *in utero* when the mother had GDM has an increased body mass index (BMI) and increased risk of developing type 2 diabetes (T2DM) [13]. These studies control as best we can in a human scenario for heritable or current lifestyle factors, and suggest an additional input from the intrauterine environment.

In this review we have focused on the impact of obesity in pregnancy on offspring outcomes. However, it is worth noting that the early post-natal period also represents a critical time window when exposures could impact on long-term health. This is particularly true in relation to metabolic health, as many neuronal connections in feeding pathways are established during this time window. Teasing out the relative effects of the *in utero* and postnatal period in humans is challenging because if a mother had obesity in pregnancy, this will likely remain into the post-natal period. Furthermore, after birth there is an additional influence of infant feeding practices on long-term cardiometabolic health [14]. Therefore, most data in relation to the distinct effects of the post-natal period on offspring health have come from rodent models and these suggest that post-natal exposure to obesity has additional effects on offspring long-term health [15]. However, due to the relative immaturity of newborn rodents compared to humans, caution should be taken with extrapolating time windows data between species.

## 2. Interaction between maternal genetics, fetal genetics and the *in utero* environment

Over the past 30 years, several large prospective birth cohorts have been conducted around the world to gain a better understanding of how the early life environment shapes future health. These studies have consistently shown positive associations between maternal BMI and glycaemia and offspring BMI [16,17]. However, such association studies cannot differentiate the relative roles of genes and the environment in mediating these effects. Recent Mendelian Randomization studies have indicated a stronger genetic component to this association than previous Multivariate analysis of the same cohorts [18]. However, it is important to note that if a mother has obesity because of genetic variants, this will impact on the fetal environment. Therefore, she can transmit the obesity to her child through both genetic and epigenetic mechanisms via the impact of the maternal genotype on the *in utero* environment (figure 1). For this reason, in Mendelian Randomization analysis it is hard to rule out violation of the exclusion restriction assumption (i.e. that maternal BMI single nucleotide polymorphisms do not influence the outcome via variables other than maternal BMI). Such studies highlight the challenges and limitations in interpreting the association between maternal BMI or adiposity and later offspring BMI and teasing out the genetic versus environmental contributions. A recent study examining maternal genetic effects on birth weight independent of fetal genetic effects reported that 7 out of the 10 maternal loci that are most strongly associated with birth weight are consistent with the maternal genotype acting via the *in utero* environment, rather than effects of shared alleles with the fetus [19]. Furthermore, a recent study has highlighted the additional impact of maternal genetics on offspring birth weight via determining gestational length [20]. Interestingly, among the known T2DM variants that influence birth weight, those with the largest effect on raising birth weight through the maternal genotype are those known to increase maternal fasting plasma glucose levels, and therefore contribute to a hyperglycaemic *in utero* environment [21]. Recent studies in humans and animal models also suggest it is maternal glucose and insulin levels that are the driving factor behind intrauterine programming rather than maternal obesity *per se* [22].

## 3. The impact of obesity in pregnancy on offspring physiology and health

### (a) Impact on birth weight and adiposity

The relationship between maternal BMI and offspring birth weight is not linear: studies report increased incidence of both large for gestational age (LGA; birth weight greater than 4000 g) babies and small for gestational age (SGA; birth weight less than 2500 g) babies from pregnancies with obesity. This is significant as both low and high birth weights are associated with childhood and adult obesity [23]. SGA is common in pregnancies with obesity that are complicated by hypertensive conditions in the mother, suggesting altered blood and therefore nutrient flow to the fetus in these pregnancies. Rapid post-natal catch-up growth after SGA birth appears to exaggerate the effect of suboptimal growth *in utero* on risk of metabolic and cardiovascular diseases later in life [24]. Indeed, early experiments in rodents demonstrated that the early post-natal growth rate is an independent risk factor for adiposity later in life [25] and there is evidence that accelerated early post-natal growth, independent of birth weight, is associated with increased later obesity [26]. By contrast to birth weight, which appears to be more strongly associated with maternal BMI than GWG, some studies have suggested that GWG is a stronger predictor of neonatal adiposity [27]. Furthermore, maternal diet during pregnancy seems to strongly influence fetal fat accretion. In both the GUSTO and Healthy Start birth cohorts, certain maternal dietary patterns (those high in carbohydrate and fat, at the expense of protein) are associated with increased newborn adiposity [28].

### (b) Impact on food intake pathways in the offspring brain

Animal models have shown that a common cause of the increased body weight observed in offspring of obese mothers is hyperphagia [29], implicating altered regulation of food intake as an underlying cause of obesity. The hypothalamus plays an essential role in maintaining energy homeostasis by coordinating food intake, energy expenditure and glycaemia. The plasticity of hypothalamic development during the perinatal period means it is susceptible to disruption by exposure to adverse environments and represents a mechanism by which changes in metabolic homeostasis can be permanently programmed in individuals. We have recently demonstrated in a mouse model that exposure to maternal obesity impacts on offspring hypothalamic development from the very early stages of development, resulting in reduced proliferation of hypothalamic neural progenitor cells in the offspring brain and early insulin resistance [30]. There is also evidence from human fetal brain imaging studies that exposure to GDM results in increased gliosis in the hypothalamus from early in gestation [31].

One of the critical periods of hypothalamic development is the generation of the neuronal projections originating in the arcuate nucleus and projecting to the paraventricular nucleus. Genetic disruption of the development of these projections in humans causes obesity [32]. These intra-hypothalamic projections are particularly susceptible to programming by maternal obesity, and a reduction in their density is often associated with hyperphagia in adulthood [33,34]. Seminal experiments by Bouret *et al.* demonstrated the requirement of leptin at precise levels and at a precise post-natal timepoint for these projections to form correctly in rodents [35]. Recent research in rodent models has shown that the timing and magnitude of post-natal leptin levels are modulated by *in utero* and post-natal nutrition [34,36]. This may explain the altered intra-hypothalamic projections and disrupted feeding behaviour observed in these animals. Indeed, artificial blockade or advancement of the post-natal leptin surge results in sexually dimorphic changes in hypothalamic neuropeptide profile and feeding behaviour in rodents, demonstrating that changes to leptin levels during the perinatal period can result in permanently disrupted feeding control.

In addition to homeostatic feeding pathways, it has also been reported that the offspring of obese mothers display alterations to reward systems in the brain that could explain the frequently reported hyperphagia. Several studies have reported programming of the mesolimbic reward system in rodent offspring, resulting in altered activation in response to feeding stimuli [37], and an increased intake of palatable food in offspring [38]. Maternal obesity has also been reported to increase the preference specifically for fatty and sugary food in offspring [37], and in a rat model the offspring of obese mothers also display increased frequency of feeding episodes, and a longer duration of feeding during a given episode [39].

### (c) Impact on the offspring heart

Altered cardiac development and function are well-documented complications of diabetic pregnancies that have been recognized since the 1940s [40]. Animal models have shown that maternal obesity during pregnancy (accompanied by impaired glucose tolerance) induces cardiac hypertrophy and causes impaired fetal cardiac function in the final trimester [41]. In humans, there is robust evidence of an association between maternal pre-pregnancy BMI and/or obesity during pregnancy, with increased incidence of congenital heart defects (CHD) in offspring [42] and premature mortality from cardiac events [43]. Unlike many other organs, cardiac development occurs during the first trimester; thus, maternal physiology during this early timeframe is relevant to the developing fetal heart. Indeed, fetal myocardial dysfunction has been detected in pregnancies complicated by obesity as early as 14 weeks of gestation [44]. Importantly, this time point is significantly before pregnant women are screened for GDM, and therefore demonstrates the importance of women entering pregnancy at a healthy BMI with adequate glycaemic control. Glucose freely crosses the placenta and it is thought that *in utero* hyperglycaemia is a major determinant of cardiac malformations, and some studies have suggested that fetal cardiac malformations are rare in obese pregnancies without GDM [42]. However, other studies have shown that the CHD increase associated with maternal obesity is significant even after adjusting for glucose levels, suggesting that abnormalities in glucose metabolism do not fully explain the risk in pregnancies with obesity [45]. As well as hyperglycaemia, hyperinsulinemia is also common in pregnancies complicated by GDM and obesity. Insulin signalling contributes to both embryonic and postnatal cardiac growth [46], and even short-term *in utero* hyperglycaemic exposure induces fetal hyperinsulinemia and rapidly induces cardiac septal overgrowth via altered myocardial proliferation in rats [47]. The success of some interventions aimed at correcting maternal hyperinsulinemia during obese pregnancies in reducing cardiac remodelling in mouse offspring (discussed in §5) suggests that glucose/ insulin signalling is key for cardiac development.

## 4. Epigenetic mechanisms in the inter-generational programming of obesity risk

The stable nature of phenotypes in offspring exposed to an *in utero* sub-optimal nutritional environment, and the reported inter-generational transmission of programming effects, suggest permanent changes in gene expression in exposed individuals. *In utero* regulation of the epigenome has received a lot of interest as a cause of these permanent, heritable changes to gene expression. DNA methylation is an essential component of normal genomic regulation. During the pre-implantation stage the embryonic genome is subjected to widespread demethylation, and then *de novo* methylation occurs at specific regions to generate a pattern of methylation that is inherited by daughter cells [48]. In siblings born before and after maternal gastric bypass surgery, significant differences in the methylation of glucose homeostasis genes were observed in white blood cell samples dependent on exposure to maternal obesity [49]. The Avon Longitudinal Study of Parents and Children (ALSPAC) study in the UK has conducted significant analysis of genome-wide methylation patterns and shown that compared with neonatal offspring of mothers with a healthy weight, a small number of CpG sites were differentially methylated in offspring of mothers with obesity, and that associations of maternal obesity with offspring methylation were stronger than associations of paternal obesity, supporting an intrauterine mechanism [50]. A larger meta-analysis within the Pregnancy and Childhood Epigenetics (PACE) Consortium, which consists of the ALSPAC cohort plus others across Europe, showed robust associations between maternal adiposity and variations in newborn blood DNA methylation, although the authors suggested some of the effects may be explained by genetic or lifestyle factors rather than an intrauterine mechanism [51]. Interestingly, a study examining cord blood methylation in pregnancies with either GDM or intra-uterine growth restriction showed significant commonality in the genes that are differentially methylated by these two differing *in utero* environments, supporting the concept that similar epigenetic modifications may underpin different prenatal exposures and underlie increased long-term risk of diseases such as T2DM [52].

Micro-RNAs (miRNAs) are small non-coding RNAs that post-transcriptionally modify target gene expression. miRNAs are a promising candidate for translating dynamic changes in nutritional state into changes in genomic regulation. A recent study in humans has shown significant differences in the expression of candidate miRNAs in circulating blood samples of newborn offspring depending on maternal pre-pregnancy BMI [53]. Studies in animals have begun to explore and demonstrate causative roles for altered miRNA expression in mediating some of the effects of maternal obesity on offspring phenotype. In brown adipose tissue, upregulation of miR-204-5p expression in offspring born to obese mothers leads to impaired adipocyte development through suppressing myogenesis and brown adipogenesis while enhancing white adipogenic commitment [54]. Our group has shown that programmed cell-autonomous upregulation of miR-126 in adipose tissue of offspring of obese mothers is responsible for downregulation of insulin receptor substrate 1 and lunapark, contributing to insulin resistance [55] and ER stress [56] in these animals. We have also shown that exposure to maternal obesity causes upregulation of miR-505-5p in the fetal hypothalamus, and downregulation of targets that are related to fatty acid sensing pathways [57]. Over-expression of miR-505-5p in the adult mouse brain causes increased intake of a high-fat diet, suggesting that programmed over-expression of this miRNA after exposure to maternal obesity could underlie altered food choices in offspring that lead to obesity.

## 5. Interventions to stop the inter-generational transmission of obesity risk

To prevent the inter-generational transmission of obesity risk, the options are to (a) ensure all mothers enter pregnancy with a healthy BMI, (b) intervene during pregnancies complicated by obesity or (c) treat after the programming has occurred but before pathology is evident. As we still lack effective strategies to reduce obesity in the general population, it is not currently feasible (or realistic) to ensure that all women enter pregnancy with a healthy BMI (although this should be a priority for future health policy—see §6). Likewise, intervening in offspring after the pathology is evident requires a greater understanding of the molecular mechanisms that mediate the effects of the *in utero* environment on offspring health, and is not yet feasible. However, pregnancy itself is a tractable timepoint for intervention as expectant mothers have increased contact with healthcare professionals.

### (a) Lifestyle interventions: exercise and diet

Lifestyle interventions are an attractive intervention route as they are minimally invasive and improve the health of both the mother and baby (and potentially other household members) at the same time. Several studies have aimed to limit GWG in women with obesity or those at risk of developing GDM through education sessions related to nutrition advice and enhanced contact with healthcare professionals throughout pregnancy. Unfortunately, to date this approach has seen limited success in either limiting GWG, or in reducing fetal macrosomia [58]. This may be because GWG only weakly associates with some of the negative outcomes of obesity in pregnancy. In view of the close association between obesity and insulin resistance, and the role that maternal insulin resistance plays in adverse pregnancy outcomes such as GDM, preeclampsia and fetal macrosomia, maternal insulin resistance may be a better primary focus for intervention studies. Physical activity is a modifiable factor that reduces insulin resistance and has the potential to reduce GWG and GDM incidence [59]. UPBEAT, a trial in the UK that provided a combined approach of dietary and exercise intervention in obese pregnancies, was successful in limiting GWG [60] and preventing cardiac remodelling in offspring at 3 years of age [61]. Although the exercise intervention was effective in reducing offspring adiposity at 6 months of age [62], this effect was no longer visible in offspring at 3 years of age [63].

Animal models have shown promising results for maternal exercise during pregnancies complicated by obesity in mitigating the negative consequences for offspring. Our laboratory has shown that in mice, a mild exercise regime during an obese glucose-intolerant pregnancy results in a rescue of maternal hyperinsulinemia that is associated with a restoration of insulin sensitivity in offspring [64], and a prevention of cardiac hypertrophy in offspring [65]. Others have similarly shown improvements in glucose tolerance, insulin levels and adiposity in male [66] and female offspring [67] of obese-exercised mothers compared to obese-sedentary mothers. Maternal exercise can also potentially overcome some of the long-term programming effects of maternal obesity on the offspring brain, by altering diet preferences, central reward system signalling [68] and hypothalamic gene expression [69]. The differences in successful outcomes of lifestyle-based interventions in humans and rodent models may stem from the fact that human adherence to any lifestyle intervention is challenging. This is particularly true in pregnancy when mothers may be affected by nausea and fatigue and therefore reluctant or unable to increase physical activity or modify diet. In addition, in human trials most interventions don't start until the second trimester when many pregnant women have their first contact with health professionals (for example, the women recruited for UPBEAT were between 15–19 weeks of gestation at enrolment in the programme), by which time some programming may have already occurred and impacted offspring outcomes.

### (b) Metformin and insulin sensitizing drugs

Metformin and sulfonylureas (SURs) are pharmacological treatments for GDM. While metformin and SURs can be equally effective in correcting maternal glycaemic control in cases of GDM, they do so via different cellular pathways, and therefore result in different neonatal outcomes. Unlike insulin, both metformin and SURs can cross the placenta. While metformin prevents fetal macrosomia, SUR treatment results in heavier neonates, probably resulting from fetal hyperinsulinemia [70]. Although primarily used for the treatment of GDM, metformin is also being trialled for pregnancies with obesity [71] due to its effectiveness in reducing gestational weight gain. Recent reports from both animal and human studies have suggested that *in utero* exposure to metformin, while effective in reducing the incidence of LGA births in GDM pregnancies, may cause fetal growth restriction [72]. Work from our group in a mouse model suggests that this may be via an impact on the placenta [73]. A recent meta-analysis of GDM pregnancies has shown that metformin-exposed neonates are significantly smaller than neonates whose mothers were treated with insulin during pregnancy [72]. This is particularly worrying due to the previously discussed link between low birth weight followed by postnatal catch-up growth and adverse long-term cardio-metabolic outcomes. Indeed, a recent study reporting the effects of metformin use in pregnancies complicated by polycystic ovaries has shown that metformin exposed children have higher BMI and increased prevalence of overweight/obesity at 4 years of age [74]. Animal studies have not yet provided a consensus regarding whether metformin administration during pregnancy causes overall beneficial [75] or detrimental [76] effects to offspring long-term metabolic health. This lack of clarity has led to the design and implementation of a new study to collate information from around the world on the efficacy and safety of metformin use in pregnancy [77].

### (c) Maternal dietary supplementation: antioxidants and methyl donors

An imbalance in the generation of reactive oxygen species and the antioxidant capacity of an organism is a known consequence of a sub-optimal early nutritional environment. Therefore, reducing cellular oxidative stress is one approach that has been adopted to prevent the detrimental effects of intra-uterine programming. In non-human primates (NHP) and rodent models, administration of the anti-oxidant drug Resveratrol to mothers with obesity during pregnancy improves maternal fasting insulin levels and decreases maternal fat mass, in addition to resolving fetal fatty liver, reducing offspring adiposity and improving leptin sensitivity and brown adipose tissue function [78,79]. Other studies in humans and rodents have targeted the reported methylation changes in offspring by supplementing the maternal diet with methyl donors, and have effectively shown changes in methylation patterns [80].

The studies described above focus on interventions during pregnancy to target fetal development. However, as discussed above, intervention to the offspring themselves is useful when intervention during pregnancy is not possible. Data from our laboratory, using the maternal low protein rat model, has demonstrated that post-weaning supplementation of the offspring diet with the antioxidant coenzyme Q10 at least in part reverses many of the consequences of nutritional programming, including effects on cardiac, hepatocyte and adipocyte ageing, inflammation, telomere shortening, DNA damage, cellular senescence and insulin resistance [81]. Furthermore, a recent study in a mouse model of maternal obesity has shown that treatment of neonatal offspring with the ER stress-relieving drug TUDCA rescues many metabolic phenotypes including increased food intake, adiposity, decreased energy expenditure and glucose and insulin intolerance [82].

## 6. Limitations and future directions

It is hard to put a figure on the relative contribution of our early life exposures in determining cardio-metabolic disease risk relative to other major contributors such as our genetics and current environment as there are complex interactions between all of these factors. The best evidence we have to date of a causal role for the *in utero* environment in programming cardio-metabolic risk is from studies examining siblings discordant for exposure to maternal obesity (where genetics and current environment are controlled as much as is feasible in a human context) and from animal models (where genetics and current environment can be tightly controlled). Furthermore, although the majority of animal studies have been undertaken in rodents, many molecular readouts reported in rodent models are also observed in NHP models of exposure to obesity in pregnancy [83]. This therefore suggests that the insight into mechanisms being gained from rodent models will be translatable to a human setting, but this remains to be further investigated.

Environmental factors are modifiable across the life course, and should therefore be a focus for interventions aimed at reducing cardio-metabolic disease incidence. Modification of the early life environment is particularly attractive as not only are individuals (pregnant women and newborns) in closer contact with healthcare professionals, but there is also the potential to benefit the health of two generations at once. The NICE guidelines for both 'Maternal and Child Nutrition' and 'Weight Management Before and During Pregnancy' are due to be updated in 2024, and this represents an important opportunity to translate some of what we have learnt in research studies into changes in clinical practice and the advice given to patients. In the UK, the all-party parliamentary group the Children's Alliance recognizes preconception care as being an underlying cause of obesity (which it lists as one of five key challenges facing young people nowadays), and in early 2023 they tabled a motion to the UK Government (https://edm.parliament.uk/early-day-motion/60537) to recognize the importance of pre-conception care and ensure that this is prioritized in policy content across all government departments.

## 7. Summary

The recent huge increase in the number of pregnancies in women who are overweight or have obesity is concerning given the increased risk of adverse outcomes that this confers, not only for immediate outcomes of pregnancy but also for the long-term cardiometabolic health of offspring. Neonatal offspring exposed to maternal obesity or GDM during pregnancy are more likely to be born with a high or low birth weight and/or increased adiposity, predisposing them to obesity later in life. Furthermore, an the intrauterine environment associated with obesity significantly impacts on the development of key organ systems such as the cardiovascular and central nervous systems. The molecular mechanisms that mediate these changes to fetal development remain largely uncharacterized, but are likely to involve regulation of the epigenome. Both lifestyle and pharmacological interventions during a pregnancy with obesity, particularly those aimed at correcting maternal insulin resistance, may be effective in limiting the inter-generational transmission of obesity susceptibility.

## Data Availability

This article has no additional data.
